# Redefining the research hospital

**DOI:** 10.1038/s41746-019-0201-2

**Published:** 2019-12-06

**Authors:** Parashkev Nachev, Daniel Herron, Nick McNally, Geraint Rees, Bryan Williams

**Affiliations:** 1 University College Hospitals NHS Trust, 235 Euston Rd, Bloomsbury, London, NW1 2BU UK; 2 Institute of Neurology, UCL, London, WC1N 3BG UK; 3 NIHR UCLH Biomedical Research Centre, Research & Development, Maple House Suite A 1st Floor, 149 Tottenham Court Road, London, W1T 7DN UK; 4 Faculty of Life Sciences, UCL, London, WC1E 6BT UK; 5 UCL Institute of Cardiovascular Sciences, UCL, London, WC1E 6BT UK

**Keywords:** Health policy, Translational research, Research management

## Introduction

All medicine was innovation, once. Yet the contemporary notion of medical research is remarkably narrow. While every clinician is encouraged to be aware of the latest advances, only a few are expected to contribute to them. Anyone may be a patient, yet clinical practice is determined by the minority included in research studies. The aim of medicine is to improve the lives of patients, yet knowledge of disease is arbitrarily prioritised as its primary means. The agents of medicine are clinicians, yet new interventions are mostly created by others, within corporate enterprise deliberately kept at arm’s length. We treat the specific, individual patient in front of us, now, yet most research is addressed to faceless, generic groups, to be realised deep into an ill-defined, hypothetical future.

These constraints arise from assumptions too widely held to be self-evident for anyone seriously to question them. It is commonly assumed, at least by those who secure funding, that clinical research must be separate from service delivery, that limited samples adequately illuminate the wider population, that improving care is conditional on enhancing explicit knowledge of biology, that translation should be devolved to industry, that a patient’s individuality is random deviation from the population mean, and that the tree of innovation bears fruit long after it blooms, if it bears any fruit at all.

**Fig. 1:**
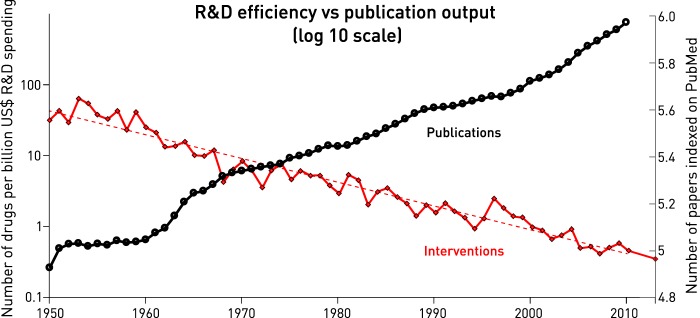
The translational crisis in medicine. Common log scale plot of the annual number of drugs approved by the FDA per billion US$ spending adjusted for inflation (in red, left *y* axis) and the annual number of articles indexed on PubMed (in black, right *y* axis) over the interval 1950–2010^1^ and 2013^2^. Note logarithmic decline of the former despite an equal and opposite rise of the latter, consistently over more than half a century.

Were medical research clearly successful, the fidelity of these assumptions would be merely academic. But measured by what really counts—the creation of real-world interventions—its productivity has been strikingly poor. The number of drugs receiving regulatory approval per billion dollars of investment has been consistently halving every nine years, while the number of medical publications has been doubling with the same periodicity (Fig. 1).^1,2^ A disinterested auditor would justifiably have shut us down a long time ago.

A failure so striking, so consistent, requires us to examine precisely the areas of universal consensus: there is nowhere else for a fault of this generality to lie. So let us take each of the foundational assumptions of clinical research, test them conceptually, and examine their ramifying consequences. We shall see the result is a very different vision of what a research hospital could—and ought—to be.

## Service and research

Each human being is unique by design—not merely construction—so every patient contact is research, whether we admit it or not. The complexities of the genetic blueprint and its interaction with the environment ensure no individual could ever be wholly predictable from any other. But each variation represents a successful biological solution that informs a local “neighbourhood” of similar solutions, and if this is true of normal physiology^3^ it will tend to be true of the disease processes that perturb it.^4^ The structure of this neighbourhood is much finer than the broad disease categories found in medical textbooks, which is why clinicians are yet to become merely agents of triage into predetermined management pathways. And it is why our practice is responsive to every patient we see, recursively adjusted throughout our professional lifetimes.^5,6^ But why should such adjustment remain tacit? If it is relevant to the practice of one clinician it is relevant to all, and ought to inform every new clinical encounter. The principal constraint on rendering it explicit—and thereby transferrable—has been the capacity of the modelling architectures on which evidence-based medicine is currently built.

Equally, service-derived intelligence need not be merely observational. That we do not randomise treatments in routine care does not mean we should not learn from the random variability that arises naturally. Yes, such variability is strongly biased by the clinician’s intentions, but this defect is nether statistically irreparable nor necessarily greater than the defects of formal studies.^7^ Every instance of an intervention is potentially informative: neglecting to attend to the most numerous—those drawn from routine care—can only strike an external observer as perverse. Crucially, escalating the scale of analysed data minimises the bias that forces us to perform independent experimental studies in the first place, solving precisely the problem sample-based studies are designed to solve, without their many limitations.^8^

## Samples and inference

The path to medical knowledge has been set for generations: we seek to infer general truths about biology and its response to clinical interventions by testing a small number of intuited hypotheses on data derived from limited samples of the population. It is traceable to 17th century beliefs in the causal simplicity of the universe, framed in 19th century statistical methods.^9^ Where the horizon of hypothesised solutions is narrow and the statistical model of each possible solution needs only a few parameters to distinguish it from the others, this is indeed a reasonable approach. But if anything is certain about biological systems it is that they are not simple.^4,10^ Biological causality only rarely operates through direct, serial pathways, with readily determinable necessary and sufficient steps. Its mechanisms are distributed across “causal fields” of multiple interacting factors, resulting in families of similar but non-identical complex biological patterns where each factor is an insufficient but necessary part of a wide set of unnecessary but sufficient conditions (so-called INUS conditionality).^11^ The fundamentally adaptive nature of biology further renders the resultant patterns unpredictable from genetics alone, for they are shaped by both chance and the history of each organism.^10^

These characteristics make the biological landscape impossible to survey through limited samples, for the terrain can only be defined by large-scale, fully-inclusive data. Across such breadth of possibility, intuition is a poor tool for selecting which hypothesis to test, and without a synoptic view over the alternatives the outcome of any empirical testing becomes moot. Furthermore, where the hypothesis space is not adequately defined talk of inference is misplaced: we cannot conclude that a given biological factor must have been the cause, only that we can predict the clinical outcome from it.^12^ For all its dominance, the practice of drawing hypothesis-driven inferences from limited samples is fundamentally faithless to biological reality: it should be no surprise that its translational yield is geometrically diminishing.

## Knowledge and impact

Medicine differs from science in being concerned with fact only as far as it has a bearing on action. Medicine, moreover, demands action even in the absence of secure knowledge, for its objective is not to demonstrate biological realities but to alter them, if necessary speculatively, in the interests of the patient it individually serves. Many critical aspects of care have little to do with biology, but instead depend on numerous procedural elements of the care pathway, including mundane logistical factors.^13,14^ If patient impact is our primary concern, innovation ought to focus on the actionable elements most likely to enhance it, regardless of their nature. For example, though stroke is supremely sensitive to the latency of treatment, it is striking that less innovation is applied to solving the procedural problem of prompt assessment than to biological problems of far more remote impact. Across London, even the trivial obstacle of identifying hospital-bound patients in advance of their arrival by ambulance is yet to be overcome, needlessly delaying their immediate management by many minutes.

Prioritising explanatory models of disease grounded in mechanistic accounts of biology neglects less ambitious models with nonetheless greater individual predictive power. Where a system is too complex to be easily explained—and our hypothesis-driven, principled models fail—data-driven, heuristic models may at least allow us to forecast the clinical outcomes of individual patients, and to adjust our management in anticipation.^15^ What would we make of a meteorologist who refused to give advance notice of a hurricane without first creating a fully explanatory model of the weather? Our belief that medicine must be more like horology than meteorology needs empirical testing, case by case, not a blanket presumption in its favour.

## Translation and enterprise

Hospitals are unlikely to become wholesale manufacturers of the interventions they exist to deliver. But that does not mean their relationship with industry must be built on the model of an agent mediating between consumer and vendor. Healthcare does not merely administer the market for interventions, it determines—professionally, not commercially—both their value and much of the biological basis for their development. So central a role is not discharged by the task of maintaining impartiality in treatment selection, and leaves open the door to much closer engagement. Indeed, as treatments become increasingly personalised, tailored to the individual patient, either more of industry would need to become embedded in hospitals, or else hospitals would need to expand into industry. The beautifully bespoke approach of chimeric antigen receptor T cell therapy,^16,17^ for example, requires a deeply-integrated, clinically-driven manufacturing process, precisely because so many of its elements are derived from patients themselves. Though still an outlier, its highly individualised design can only become the norm, not just in oncology but across the whole of medicine, for it is precisely this characteristic that makes the therapy so disruptively effective.

Clinical interventions are only one part of healthcare for which we must lean on industry. All intervention is preceded and accompanied by clinical investigation, which in common with all forms of intelligence-gathering relies on tools for acquiring and organising information. Traditionally subcontracted to companies with limited clinical knowledge, healthcare information technology—especially electronic patient record systems—is often ill-suited to its purpose. Yet few of the many clinicians who criticise the technological standard here have attempted to redefine it, and none of the professional bodies that are supposed to give us all a unified voice. As the principal consumers of healthcare information technology it is strange that hospitals should be so disengaged from the process of creating it.

## Individuals and groups

**Fig. 2:**
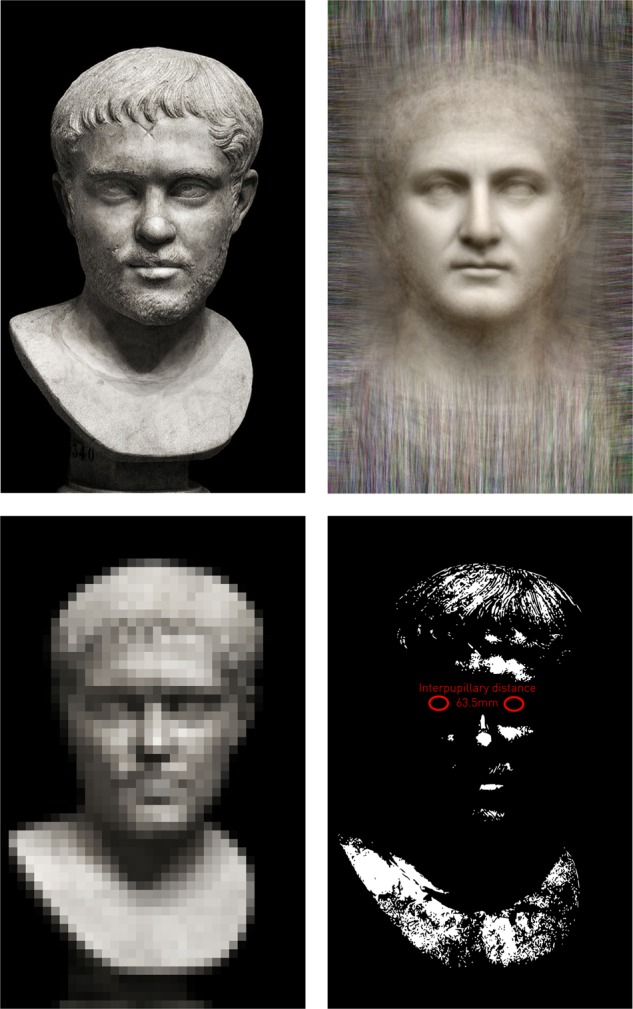
Individuality and dimensionality. The face of the Roman Emperor Hostilian (top left) is poorly described by the canonical face of all Roman Emperors (top right), which is—by definition—not identical with any of the individual faces from which it is derived. Furthermore, the individuality of a face is better captured by a low-precision, high-dimensional parameterisation (bottom left), than it is by a high-precision, low-dimensional parameterisation such as the inter-pupillary distance (bottom right). The photograph of Hostilian is reproduced with the kind permission of Dr William Storage.

Contemporary talk of “personalised medicine” must strike an outsider as odd: to whom, if not the individual person, could medicine have been addressed all this time? We study groups only because we have no other means of drawing inferences about the individuals who have always been our primary concern. But the historical failure of this approach to illuminate the care of the individual patient—a failure conceded in speaking of personalised care as an innovation—is not a consequence of drawing intelligence from large groups. The problem is the insistence on modelling biology within explanatory models with few variables, applied to groups that are, if anything, far too small. The reality literally staring us in the face is that individuality is defined by a multiplicity of many variables, intelligible only within complex, high-dimensional multivariate models, and cannot be captured by any simple set of “biomarkers” without breaking the laws of physics (Fig. 2).^18^ If adequately individuating models of the face, a part of the body encoded by relatively few genetic loci,^19^ require a high-dimensional specification, what hope can there be for the simple models so widely pitched at conditions we know are genetically vastly more polymorphous? Our attempts to find simple, elegant, “mechanistic” explanations for disorders of great population impact such as heart disease, stroke, diabetes, and dementia are likely to be failing not because we are yet to find the correct explanation but because we are looking for fundamentally the wrong kind.

The stark, information theoretically-prescribed reality is that the holy grail of personalised medicine will never be found if we insist on looking at the biological world through simple, reductive, biomarker-filtered glasses. And those of us who promise to deliver it should realise they are committing themselves to a high-dimensional vision of medicine, and to all that follows in inevitable consequence.

## The future, now

Until a decade ago, the position presented here would have been too nihilistic to be worth defending. The price of making medicine rigorous, reproducible, scientific, was to accept its object could only ever be rendered in rough caricature. But the evolution of our inferential tools has lagged far behind the richness of our investigational instruments. A routine magnetic resonance scan of the brain contains hundreds of thousands of variables, and a district general hospital of modest size will have many thousands of such studies lying in storage. A typical general medical admission will be chronicled by a rich time series of blood tests, conveyed and stored in digital form for decades. The clinical narrative, even in the form of prose clinic letters and unstructured discharge summaries, records a wealth of information contemporary natural language processing algorithms can render analysable at large scale. The individuating richness of detail is already there in the routine clinical stream: what has been missing is the mathematical and computational means of harnessing it within high-dimensional models powered by large-scale data.

The rise of machine learning has changed all that.^20^ Though by no means trivial, the introduction of high-dimensional modelling within the clinical stream is universally achievable across the entire domain of medicine, and inexpensive compared with conventional research taking into account the cost of data acquisition, here naturally borne by the clinical service itself. This is a future we could have now, if we chose to pursue it. Its impact will vary from one application to the next, but it would be hard to conceive of a broader, more fundamental, catalyst of innovation, or one more obviously—and desperately—needed.

## An alternative vision

These considerations suggest a radical revision of what a research hospital ought to be. We need not discard the conventional, “classical” research programmes mature hospitals accommodate with practised ease, but enable a wholly new approach in parallel. It is not simply about implementing the now familiar idea of a “learning hospital”, continuously optimised by the clinical stream that flows through it.^21,22^ Rather, it is about what a hospital learns, how it learns it, and what it does with the intelligence it acquires.

If the biology of each patient requires a multitude of variables to characterise, we must model as many as we collect, for even the maximum will be suboptimal. Since the importance of a variable may be manifest only in its complex interaction with others, it cannot be established with simple linear models run on modest samples of data. What factors are material to an outcome of interest, or any other actionable aspect of care, should be determined by comparison of models beginning with all the facts available to us, not an arbitrarily small, “audited” subset. Nor may we assume that any predictive pattern we identify will remain static, or be generalisable across all sub-populations. We need models of all the data, from all patients, all of the time.

If the difficulty of the problem, so formulated, raises concerns about its only plausible solution—the stability, generalisability, interpretability and equitability of high-dimensional models—we should calibrate our alarm against the manifest defects, in each of these respects, of conventional, “gold standard” evidence-based medicine. If we are worried about the stability of a complex adaptive model faced with non-stationary data, we should be even more worried about simple models that blithely assume stationarity. The generalisability of low-dimensional models is typically an artefact of their poor fit: it is less a virtue than cashback from an arguably greater vice. The interpretability of any model must be dictated by the nature of the problem, not the satisfaction of our intellectual vanity: if no good simple model can be found, then a complex model is the best solution, whether we like it or not. And if we are worried about a model underperforming within a specific community, raising ethical concerns about equitability, we should remember that any low-dimensional model is constitutionally biased against each individual patient in direct proportion to his or her distance from the population mean.

The introduction of machine learning does not create these difficulties but brings them out of shadows—cast by evidence-based medicine—that have concealed them for far too long. Yes, we need enhanced infrastructure—clinical, conceptual, computational, and regulatory—but the need is independent of the style of modelling it supports, and can be satisfied through organic growth of the governance systems already in place. A sharp distinction must be drawn between the use of complex modelling as an instrument for individualised evidential synthesis—operated by human experts—versus as a cheap substitute for human expertise it could only approach asymptotically. A key role for such infrastructure is to stimulate and guide the former while powerfully inhibiting the latter.

Who would pay for all of this, one might ask, on so speculative a prospectus? And how would such an approach translate globally, across enormous variations in healthcare resourcing? To the efficiency of working on data that is already collected, stored, and partially curated—for example, the MR brain imaging catalogue of our hospital trust alone would cost ~£100 million to replicate—we should add the substantial value of complex analytics in enabling operational optimisation currently obstructed not by a lack of organisational skill, but by the limits of simple solutions in the face of medicine’s constitutionally extreme heterogeneity. Even something as fundamental as scheduled hospital attendance can be greatly improved with relatively lightweight machine learning, accelerating care and improving equitability of access while saving ~£2 to £3 per appointment.^23^ Better patient care here need not come at a cost: quite the opposite. And the rapid commodification of the digital realm on which machine learning depends, coupled with the globalisation of connectivity, means the approach need not be limited to wealthy nations: indeed, it is potentially a potent, rapidly distributable leveller of standards of care.

An operational focus need not inhibit clinical applications, indeed it is an essential catalyst of their development. A high-dimensional model predicting a given outcome—mobility in stroke, for example—may have both clinical and operational uses: the former concerned with the management of the individual patient, the latter with the management of the clinical unit as a whole. But the latter use allows us to draw benefit from an implementation while quantifying and certifying its clinical utility at scale and with appropriate data inclusivity. Such models can also operate at any time scale, dynamically optimising care with a temporal granularity of minutes or hours, not the many months typical of audit-driven improvement. Again, fast optimisation of this kind does not imply excluding human experts from the decision chain, but rather accelerating the presentation of evidence on which humans will always ultimately take all critical decisions.

A vision of such digital sophistication may seem a wild hallucination in a contemporary health service environment still learning 90 s technology most of us have already forgotten. Even those persuaded of its merits are tempted to turn to industry to deliver it, viewing hospitals as crude miners of a new commodity—healthcare data—it is for others to turn into medical gold. The dark glamour of “artificial intelligence”—the haute couture of enterprise—reinforces a common belief this is luxury technology only very special companies can deliver, either at exorbitant financial cost, or with the concession of collaterally using the data for less sympathetic ends. This belief is mistaken. A problem so central to healthcare can be no more outsourced than history-taking or clinical examination. This is a capability to be built internally—in collaboration with academia and industry, of course—understood and controlled as only an organic part of the organisation can be. Yes, it requires a seismic cultural and intellectual shift, but neither the digital infrastructure nor the conceptual tools of machine learning are beyond clinicians to acquire.

Indeed, machine learning is not speculatively progressive but comfortingly regressive: a return to the traditional clinical focus on the individual patient, richly characterised, that “evidence-based medicine” took us away from. Yet it is not a departure from evidence-based medicine but its reformulation in individually-centred form, combining some of the rich complexity of clinical intuition with all the formal rigour of traditional statistics. We can finally have our cake and eat it.

## Data Availability

The data generated and analysed in the current report are available from the corresponding author on reasonable request.
